# Association of visual acuity with sleep quality and sleep duration in patients with type 2 diabetes: evidence from a cross-sectional analysis of the Fushun Diabetic Retinopathy Study

**DOI:** 10.3389/fpsyt.2025.1521347

**Published:** 2025-07-17

**Authors:** Yu Wang, Shisong Rong, Zixi Zhou, Yuanbo Liang, Zhong Lin, Fenghua Wang, Qitong Wang, Kemi Feng, Xiaoxia Ding, Dongxiao Zang, Dong Li, Bo Zang

**Affiliations:** ^1^ Department of Ophthalmology, Fushun Eye Hospital, Fushun, Liaoning, China; ^2^ Mass Eye and Ear, Mass General Brigham, Harvard Medical School, Boston, MA, United States; ^3^ Eye Hospital, Wenzhou Medical University, Wenzhou, China; ^4^ Department of Ophthalmology, Beijing Tongren Eye Center, Beijing Ophthalmology & Visual Science Key Laboratory, Beijing Tongren Hospital, Capital Medical University, Beijing, China; ^5^ Department of Ophthalmology, China Medical University, Shenyang, Liaoning, China

**Keywords:** visual acuity, sleep quality, sleep duration, Pittsburgh Sleep Quality Index (PSQI), cross-sectional study

## Abstract

**Objective:**

To examine the association between visual acuity (VA), sleep quality, and sleep duration among Chinese adults.

**Subjects/Methods:**

Data were derived from the Fushun Diabetic Retinopathy Cohort Study (FS-DIRECT), a cross-sectional, community-based study conducted in Fushun, China, from July 2012 to May 2013. The study included 1284 participants (58.7% female, mean age 61.3 years) with type 2 diabetes. VA was assessed using the LogMAR chart and stratified into six groups based on LogMAR scores: <0 (optimal), 0-0.1, 0.1-0.2, 0.2-0.3, 0.3-0.5, and ≥0.5 for multivariable-adjusted analyses. Sleep quality was evaluated using the Pittsburgh Sleep Quality Index (PSQI).

**Results:**

Restricted cubic splines revealed a significant J-shaped association between VA and sleep quality (*P* for non-linearity = 0.004). This relationship was characterized by a positive correlation for VA values below 0.5 LogMAR, with the association plateauing at higher VA levels. Compared to the optimal VA group, the odds ratios (ORs) for poor sleep quality were: 1.18 (95% CI: 1.05-1.33) for 0-0.1, 1.73 (1.52-1.97) for 0.1-0.2, 1.99 (1.74-2.28) for 0.2-0.3, 1.55 (1.33-1.80) for 0.3-0.5, and 1.80 (1.54-2.10) for ≥0.5. No significant association was found between VA and sleep duration, whether short or long, in fully adjusted models.

**Conclusions:**

Our findings reveal a J-shaped association between visual acuity and sleep quality, while no significant association was observed with sleep duration. Even mild vision changes, not typically classified as impairment, may significantly affect sleep. This underscores the importance of early sleep health consideration in eye care.

## 1 Introduction

Sleep disorders affect over 1 billion people worldwide (1). Poor sleep quality is linked to cardiovascular diseases, metabolic disorders, cognitive impairments, and increased mortality risk, thus constituting an integral component of holistic health (2, 3). Individuals with visual impairment experience sleep disturbances at rates 2–5 times higher than the general population (4, 5). This problem is particularly pronounced in patients with diabetes mellitus (DM). These patients face a dual challenge: high prevalence of visual impairment (6), affecting 21.7% of patients due to diabetic retinopathy and other complications, and high prevalence of sleep disorders (7), affecting 55-86% of patients, including obstructive sleep apnea and insomnia. However, despite the clinical significance of this relationship, a critical research gap remains. No studies have systematically examined the associations between visual acuity (VA), sleep quality, and sleep duration in diabetic populations while considering diabetic retinopathy staging as a potential modifying factor (8).

Visual impairment may affect sleep quality through reduced light input disrupting circadian rhythms (9) and dysfunction of photoreceptor cells including intrinsically photosensitive retinal ganglion cells (ipRGCs) (10). Few studies have examined the relationship between visual acuity (VA) and sleep quality (11–14). Most existing research has focused on patients with visual impairment (VI), overlooking individuals with VA decline that does not meet clinical VI thresholds. Furthermore, the effects of varying degrees of VA decline on sleep quality remain poorly understood.

The Fushun Diabetic Retinopathy Cohort Study (FS-DIRECT) (15) offers a unique opportunity to bridge existing research gaps. We specifically focused on patients with type 2 diabetes as this population has a higher prevalence of both visual impairment and sleep disturbances. We aimed to examine whether visual acuity affects sleep quality even during mild visual decline before reaching clinical visual impairment thresholds, and whether the association between VA and sleep parameters in diabetic populations is independent of diabetic retinopathy status.

We hypothesized that (1): decreased visual acuity would be associated with poorer sleep quality and altered sleep duration, with this relationship being non-linear and potentially exhibiting threshold effects; (2) the association would be modified by diabetic retinopathy severity; and (3) even mild visual decline not meeting clinical visual impairment criteria would significantly impact sleep parameters.

## 2 Materials and methods

### 2.1 Participants and design

Originating from the FS-DIRECT, our research team conducted a community-based cross-sectional study from July 2012 to May 2013. We meticulously screened type 2 diabetes mellitus (T2DM) patients from 15 communities in Fushun, Liaoning Province, China, for inclusion. A thorough methodology pertinent to this study has been delineated in a preceding publication (15). Briefly, eligible participants underwent a comprehensive assessment comprising clinical, biochemical, anthropometric, and ocular examinations, along with questionnaires. Trained interviewers thoroughly collected data on socio-demographic details, self-reported medical histories, and current pharmacological treatments. Following the Declaration of Helsinki, the Ethical Committee of Fushun Eye Hospital granted ethical approval (FSYBLL-2012-01). Written informed consent was obtained from all participants. Those with inconclusive VA tests or incomplete Pittsburgh Sleep Quality Index (PSQI) questionnaires were excluded.

### 2.2 Visual acuity testing and definition of visual impairment

In line with the Early Treatment Diabetic Retinopathy Study guidelines, participants’ VA was assessed using the logMAR chart (Precision Vision, USA) from a standard distance of 4 meters. For those unable to meet the reading benchmarks, VA tests were adjusted to 1 meter. VA quantification was based on the number of letters correctly identified, assigning logMAR values for reduced vision (16). Automated refraction was performed with the NIDEK AR-610/630A Automatic Refractor (Japan). An experienced optometrist conducted a detailed subjective refraction, determining the best-corrected visual acuity (BCVA). According to US criterion guidelines, a BCVA in the better eye ≤ 20/40 is classified as VI (17, 18).

### 2.3 Assessment of DR

DR was assessed using 6-field fundus photography. The grading protocols for DR were based on the Early Treatment Diabetic Retinopathy Study (ETDRS) adaptation of the modified Airlie House classification of DR (19). The following criteria were used for grading the eyes: mild to moderate non-proliferative DR (NPDR) was characterized as levels 31-47; severe NPDR (levels 53) and proliferative DR (PDR) encompassed levels 60-85.

### 2.4 Assessment of sleep quality and duration

Sleep quality was assessed using the Chinese version of the PSQI questionnaire (20). This esteemed instrument, widely recognized in both clinical and research realms (21), offers insights into an individual’s subjective sleep quality over the recent month. It comprises 18 items across seven domains: subjective quality of sleep, sleep latency, sleep duration, habitual sleep efficiency, sleep disturbance, use of sleep medication, and daytime dysfunction (22). Each domain is scored from 0 to 3, leading to a total PSQI score ranging from 0 to 21, with higher scores indicating poorer sleep quality (22). A total score of 5 or lower suggests good sleep quality, while scores above 5 indicate poor sleep quality (21–23). For sleep duration assessment, Component 3 of the PSQI, which measures the number of hours of nighttime sleep, was utilized. Sleep durations were categorized as follows: less than or equal to 6 hours per night as short, more than 8 hours as long, and 6 to 8 hours as normal (24).

### 2.5 Statistical analyses

T-tests and χ^2^ tests were utilized to compare baseline characteristics. Binary logistic regression was used to calculate odds ratios (ORs) and their 95% confidence intervals (CIs) for assessing the association between VA and sleep quality, as well as long and short sleep duration. VA was categorized into six LogMAR-based groups: <0 (Group 1: the best), 0-0.1 (Group 2), 0.1-0.2 (Group 3), 0.2-0.3 (Group 4), 0.3-0.5 (Group 5), and ≥ 0.5 (Group 6: the worst). We also assessed the impact of a per 2-line VA decrease, corresponding to a per 0.2 increase in LogMAR units, indicative of a clinically meaningful deterioration in vision. We constructed two statistical models, accounting for potential sociodemographic and biochemical confounders. Model 1 represented the unadjusted analysis, whereas model 2 was adjusted for age (continuous), sex, marital status (unmarried/married), educational level (< 12 years/≥ 12 years of education), duration of diabetes (continuous), hemoglobin A1c (continuous), body Mass Index (continuous), and the prevalence of medical conditions like stroke, chronic kidney disease (CKD), hyperlipidemia and coronary heart disease (CHD). Stemming from Model 2, we incorporated restricted cubic splines (RCS) with three knots in our analysis of VA, aiming to delineate the nature of its associations with sleep quality and duration. In instances of non-linearity, a two-part piecewise linear model with a single change point was estimated. This involved evaluating all possible change point values and choosing the one that provided the highest likelihood. In sensitivity analyses, we imputed missing values of covariates using multiple imputation with predictive mean matching (PMM) to evaluate the potential impact of incomplete data on the relationships between VA and sleep patterns. Analyses were conducted across five distinct imputed datasets, with results synthesized in accordance with Rubin’s guidelines. To bolster the validity of results derived from model 2, we also incorporated considerations for additional potential confounders: employment (unemployed/employed), annual household income (<36,000/≥36,000 yuan), behavioral tendencies like smoking status (never, former, current) and alcohol intake (never, former, current). Furthermore, treatment of diabetes (none, oral medication, insulin therapy) were factored in, along with ocular diseases, including age-related macular degeneration (AMD), glaucoma, and optic nerve disorders, and severity of diabetic retinopathy (DR) graded as: no DR, mild non-proliferative diabetic retinopathy (NPDR), moderate NPDR, severe NPDR, and proliferative diabetic retinopathy (PDR). We tested whether age, sex, marital status, educational level, and duration of diabetes modified the association of VA with sleep quality and duration with a multiplicative interaction term. Findings from secondary and subgroup analyses, due to potential type I error, are considered exploratory. All analyses were executed using R software version 4.3.1, at a significance threshold of 0.05 (2-sided).

## 3 Results

### 3.1 Study population characteristics

**Figure 1:**
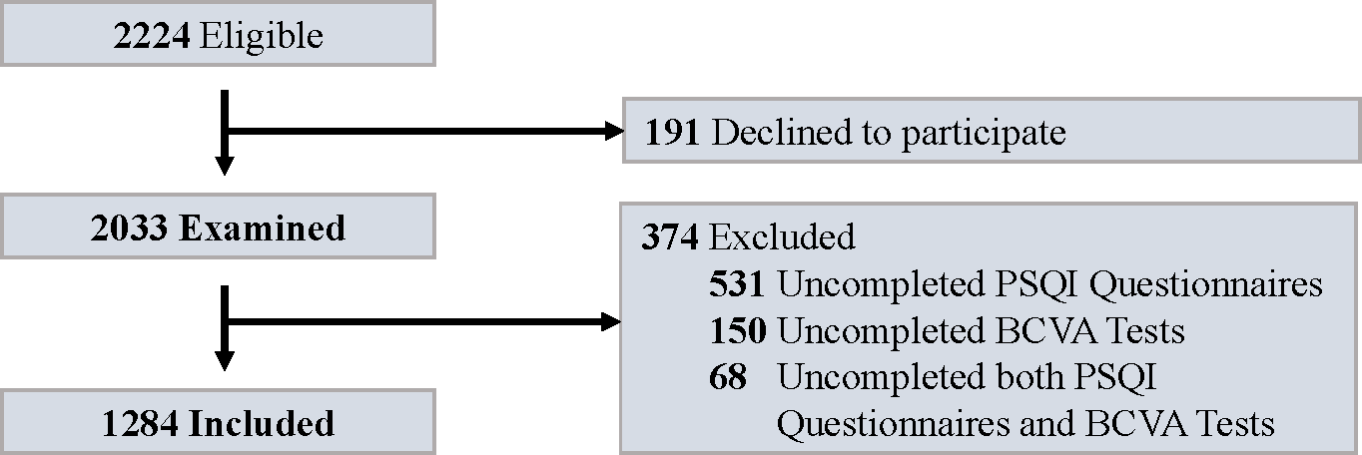
Flowchart for study enrollment. PSQI, Pittsburgh Sleep Quality Index; BCVA, best-corrected visual acuity.

**Figure 1** outlines the recruitment process for study participants. Among the 1,284 individuals evaluated, 530 (41.3%) were male, and 754 (58.7%) were female. The cohort’s mean age was 61.3 years, with a standard deviation of 8.6 years. Of these, 784 (61.1%) reported good sleep quality, while 500 (38.9%) experienced poor sleep quality. Short sleep duration was reported by 523 (40.7%) participants, and long sleep duration by 116 (9.0%).

**Table 1** indicates that participants with poor sleep quality were more often female, less frequently married, had lower educational levels, a longer duration of diabetes, and were more likely to have a history of CHD, compared to those with good sleep quality (all *P* ≤ 0.03). Distinctions in age, income, and other comorbidities between the two groups were not statistically significant (all *P* ≥ 0.05).

**Table 1:** Demographic and clinical characteristics stratified by sleep quality.

Characteristic	No. (%)		*P* value
	Good sleep quality^a^ (n = 784)	Poor sleep quality^a^ (n = 500)	
Age, mean (SD), y	60.9 (8.5)	62.0 (8.8)	0.025
Sex			0.001
Male	351 (44.8)	179 (35.8)	
Female	433 (55.2)	321 (64.2)	
Marital status			0.005
Unmarried	95 (12.1)	89 (17.8)	
Married	689 (87.9)	411 (82.2)	
Education level			<0.001
<12 y	434 (55.4)	333 (66.6)	
≥12 y	349 (44.5)	166 (33.2)	
Do not know/missing	1 (0.1)	1 (0.2)	
Annual household income, yuan			0.05
<36000	274 (34.9)	202 (40.4)	
≥36000	503 (64.2)	295 (59.0)	
Do not know/missing	7 (0.9)	3 (0.6)	
Employment			0.88
Unemployed	675 (86.1)	432 (86.4)	
Employed	109 (13.9)	68 (13.6)	
Smoking status			0.31
Never	478 (61.0)	326 (65.2)	
Former	129 (16.5)	72 (14.4)	
Current	177 (22.6)	102 (20.4)	
Alcohol intake			0.51
Never	553 (70.5)	346 (69.2)	
Former	57 (7.3)	31 (6.2)	
Current	174 (22.2)	123 (24.6)	
Duration of diabetes, mean (SD), y	7.36 (5.78)	8.20 (6.18)	0.014
HbA1c, Mean (SD), %	7.7 (1.8)	7.9 (2.1)	0.069
Treatment of diabetes			0.56
None	77 (9.8)	40 (8.0)	
Oral medication	482 (61.5)	310 (62.0)	
Insulin therapy	189 (24.1)	124 (24.8)	
Do not know/missing	36 (4.6)	6 (1.2)	
BMI, mean (SD), kg/m2	26.6 (3.2)	26.4 (3.6)	0.26
Hypertension			0.63
No	445 (56.8)	288 (57.6)	
Yes	316 (40.3)	193 (38.6)	
Do not know/missing	23 (2.9)	19 (3.8)	
Coronary heart disease			<0.001
No	536 (68.4)	287 (57.4)	
Yes	212 (27.0)	186 (37.2)	
Do not know/missing	36 (4.6)	7 (1.4)	
Stroke			0.69
No	638 (81.4)	396 (79.2)	
Yes	135 (17.2)	89 (17.8)	
Do not know/missing	11 (1.4)	15 (3.0)	
Chronic kidney disease			0.11
No	605 (77.2)	407 (81.4)	
Yes	171 (21.8)	91 (18.2)	
Do not know/missing	8 (1.0)	2 (0.4)	
Hyperlipidemia			0.32
No	390 (49.7)	236 (47.2)	
Yes	386 (49.2)	262 (52.4)	
Do not know/missing	8 (1.0)	2 (0.4)	

BMI, body mass index; HbA1c, hemoglobin A1c.

a Sleep quality categories are defined by a score of less than 5 for Good Sleep Quality and a score of 5 or greater for Poor Sleep Quality according to the Pittsburgh Sleep Quality Index.

### 3.2 Sleep parameters in visual impairment and non-visual impairment groups

**Table 2** indicates that the VI group had a higher prevalence of poor sleep quality, with 75 individuals (49.0%) affected, compared to 425 individuals (37.6%) in the non-VI group (*P* = 0.006). The average PSQI total score was significantly higher in the VI group (6.2 ± 3.8) than in the non-VI group (5.3 ± 3.4; *P* = 0.002). Notable differences were observed in specific PSQI subscales: individuals with VI had higher scores in sleep latency (1.6 ± 1.1 vs. 1.4 ± 1.0; *P* = 0.02), sleep disturbances (1.3 ± 0.6 vs. 1.2 ± 0.6; *P* = 0.02), and daytime dysfunction (0.4 ± 0.8 vs. 0.2 ± 0.5; *P* < 0.001).

**Table 2:** PSQI domain scores in patients with and without visual impairment.

PSQI	Mean (SD)			*P* value
	All (n = 1284)	No VI (n = 1131) ^a^	VI (n = 153) ^a^	
Subjective sleep quality	1.0 ± 0.8	1.0 ± 0.8	1.1 ± 0.8	0.13
Sleep latency	1.4 ± 1.0	1.4 ± 1.0	1.6 ± 1.1	0.023
Sleep duration	0.7 ± 1.0	0.7 ± 0.9	0.8 ± 1.1	0.18
Habitual sleep efficiency	0.8 ± 1.1	0.7 ± 1.1	0.9 ± 1.2	0.17
Sleep disturbance	1.2 ± 0.6	1.2 ± 0.6	1.3 ± 0.6	0.015
Use of sleep medication	0.1 ± 0.5	0.1 ± 0.5	0.1 ± 0.5	0.52
Daytime dysfunction	0.2 ± 0.6	0.2 ± 0.5	0.4 ± 0.8	<0.001
Total score	5.4 ± 3.5	5.3 ± 3.4	6.2 ± 3.8	0.002
Poor sleep quality, No. (%)^b^	500 (38.9)	425 (37.6)	75 (49.0)	0.006
short sleep duration, No. (%)^c^	523 (40.7)	460 (40.7)	63 (41.2)	0.91
long sleep duration, No. (%)^d^	116 (9.0)	97 (8.6)	19 (12.4)	0.12

PSQI, Pittsburgh Sleep Quality Index; VI, visual impairment.

a VI is defined as a best-corrected visual acuity of 20/40 or worse in the better-seeing eye.

b Poor sleep quality is defined as a PSQI total score greater than 5.

c Short sleep duration is defined as 6 hours or less of sleep per night.

d Long sleep duration is defined as more than 8 hours of sleep per night.

### 3.3 Association between visual acuity and sleep quality

**Figure 2:**
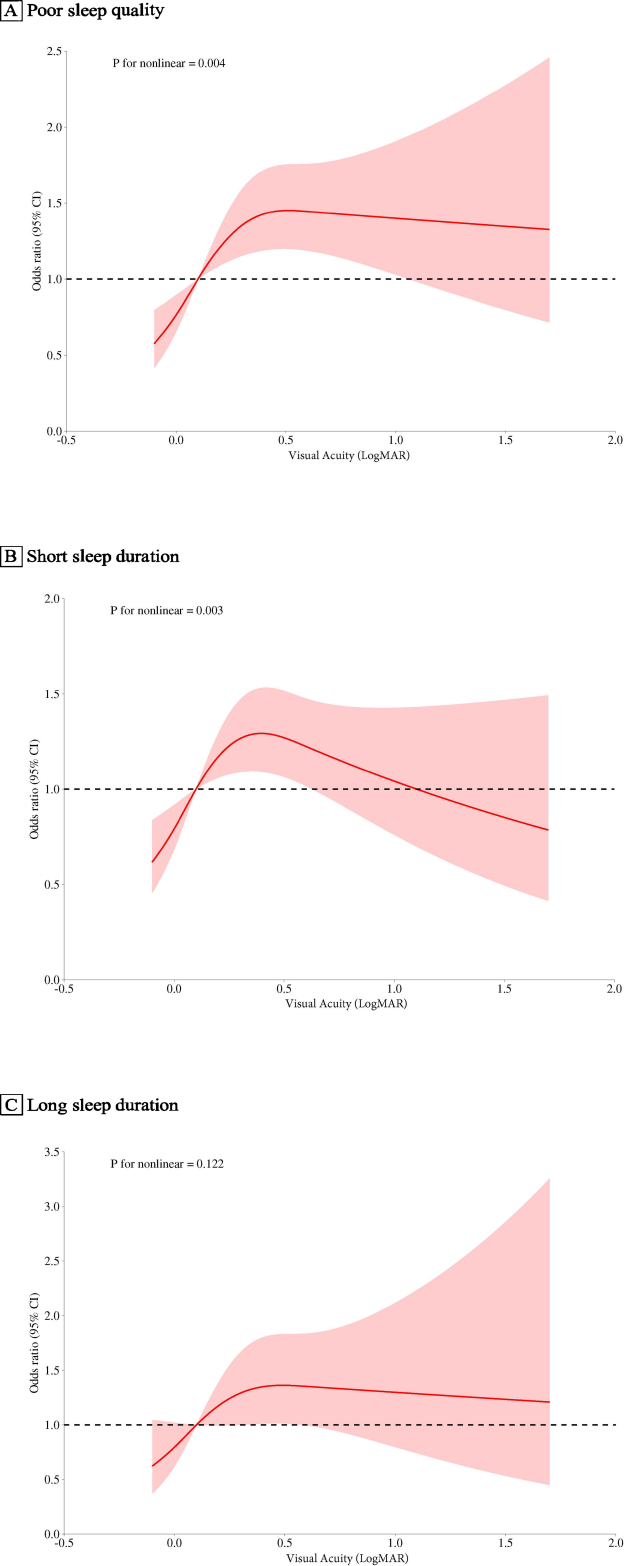
Associations of Visual Acuity with Sleep Outcomes in the FS-DIRECT Study **(A)**, For poor sleep quality vs. good sleep quality. **(B)**, For short sleep duration vs. normal sleep duration. **(C)**, For long sleep duration vs. normal sleep duration. The red solid line represents the estimated odds ratios, and the shaded area indicates the 95% confidence intervals, with visual acuity (LogMAR set at 0.0 as a reference, denoted by pink vertical lines). Covariates included age, sex, marital status, educational level, duration of diabetes, hemoglobin A1c, body Mass Index, and the prevalence of medical conditions like stroke, chronic kidney disease, hyperlipidemia and coronary heart disease. OR, odds ratio.

Elevated VA, measured in LogMAR, showed a positive correlation with an increased odds ratio (OR) for poor sleep quality. Specifically, each two-line decline in VA resulted in an OR of 1.133 (95% CI: 1.063 to 1.208) in the unadjusted model 1. Compared to Group 1 (optimal VA), ORs for Groups 2 to 6 were 1.276 (0.957 to 1.706), 1.929 (1.208 to 3.078), 2.485 (1.445 to 4.296), 1.844 (1.121 to 3.023), and 2.108 (1.424 to 3.126). Although these correlations slightly diminished following further analysis in the fully adjusted model 2, the trend remained consistent (**Table 3**). A non-linear relationship was detected between VA and sleep quality (*P* for non-linearity = 0.004), with a strong positive correlation for VA below approximately 0.5, with little evidence of association at higher VAs (**Figure 2A**).

**Table 3:** Associations of visual acuity with sleep quality and duration.

Sleep outcome and model analysis	OR (95% CI)^a^							*P* value for trend
	Group 1: (<0 LogMAR)	Group 2: (0-0.1 LogMAR)	Group 3: (0.1-0.2 LogMAR)	Group 4: (0.2-0.3 LogMAR)	Group 5: (0.3-0.5 LogMAR)	Group 6: (≥0.5 LogMAR)	Per 0.2 LogMAR increase	
Poor sleep quality								
No.	332	560	94	64	81	153	1284	
Model 1^b^	1 [Reference]	1.276 (0.957, 1.706)	1.929 (1.208, 3.078)	2.485 (1.445, 4.296)	1.844 (1.121, 3.023)	2.108 (1.424, 3.126)	1.133 (1.063, 1.208)	<0.001
Model 2^c^	1 [Reference]	1.167 (0.864, 1.581)	1.752 (1.072, 2.861)	2.006 (1.130, 3.574)	1.475 (0.868, 2.497)	1.698 (1.111, 2.598)	1.089 (1.017, 1.167)	0.011
Short sleep duration								
No.	308	510	86	60	70	134	1168	
Model 1^b^	1 [Reference]	1.167 (0.897, 1.520)	1.426 (0.941, 2.165)	1.658 (0.998, 2.770)	1.547 (0.956, 2.510)	1.241 (0.855, 1.801)	1.024 (0.965, 1.086)	0.199
Model 2^c^	1 [Reference]	1.181 (0.894, 1.562)	1.371 (0.881, 2.135)	1.541 (0.895, 2.661)	1.465 (0.875, 2.459)	1.177 (0.783, 1.770)	1.003 (0.940, 1.069)	0.510
Long sleep duration								
No.	213	327	52	34	45	90	761	
Model 1^b^	1 [Reference]	1.255 (0.773, 2.081)	1.181 (0.501, 2.564)	0.938 (0.265, 2.594)	2.382 (1.083, 5.019)	1.691 (0.883, 3.185)	1.059 (0.959, 1.159)	0.078
Model 2^c^	1 [Reference]	1.259 (0.755, 2.142)	1.180 (0.483, 2.675)	0.845 (0.229, 2.478)	2.265 (0.971, 5.093)	1.482 (0.727, 2.971)	1.019 (0.912, 1.126)	0.261

OR, odds ratio.

a The odds ratios for poor sleep quality vs. good sleep quality, short sleep duration vs. normal sleep duration, and long sleep duration vs. normal sleep duration in logistic regression.

b Unadjusted model.

c Model adjusted for age, sex, marital status, educational level, duration of diabetes, and coronary heart disease.

### 3.4 Association between visual acuity and sleep duration

Regarding short sleep duration, a two-line decrease in VA was associated with an OR of 1.024 (95% CI: 0.965 to 1.086) in model 1. ORs for VA Groups 2 to 6, compared with Group 1, were 1.167 (0.897 to 1.520), 1.426 (0.941 to 2.165), 1.658 (0.998 to 2.770), 1.547 (0.956 to 2.510), and 1.241 (0.855 to 1.801). In Model 2, these relationships were slightly reduced, with none achieving statistical significance. A significant non-linear correlation (*P* for non-linearity = 0.003) was observed, indicating positive associations with VAs below approximately 0.4, reversing at higher values (**Figure 2B**).

For long sleep duration, an OR of 1.059 (95%CI: 0.959 to 1.159) for each two-line reduction in VA was observed in Model 1. ORs comparing groups 2 through 6 of VA with group 1 were as follows: 1.255 (0.773 to 2.081), 1.181 (0.501 to 2.564), 0.938 (0.265 to 2.594), 2.382 (1.083 to 5.019), and 1.691 (0.883 to 3.185). After further adjustments in Model 2, these correlations became less pronounced. Notably, no significant linear (*P* for trend = 0.078) or non-linear (*P* for non-linearity = 0.122, **Figure 2C**) relationships were found between VA and long sleep duration.

### 3.5 Sensitivity and subgroup analyses

Subsequent sensitivity analyses showed little impact on the outcomes (**Supplementary Table 1**). Subgroup analyses revealed that VA was more strongly associated with sleep quality among older individuals, females, the unmarried, those with lower education levels, and those with a longer duration of diabetes. However, as indicated in **Supplementary Figure 1**, no significant interactions were found between these covariates and VA in relation to either sleep quality or duration.

## 4 Discussion

In our investigation, we observed that diminished VA correlates significantly with poorer sleep quality, particularly affecting aspects such as sleep latency, sleep disturbances, and daytime dysfunction. Notably, no significant association was discerned between VA and sleep duration, whether short or long.

These findings align with several previous studies. Earlier research has shown a connection between VI and poor sleep quality (12, 14), specifically affecting sleep latency (25), premature morning arousals, sleep disturbances (25, 26), and daytime dysfunction (25). However, it is pertinent to note that these studies predominantly relied on self-reported VI or utilized non-standardized sleep questionnaires. Additionally, many failed to adjust for potential confounding variables, which could influence the observed associations.

A contributing factor to sleep disturbances associated with declined VA could be reduced light input, often resulting from ocular diseases like cataracts. Light is instrumental in the synchronization of homeostatic and circadian rhythms, crucial for the optimization of sleep patterns (9). The reduced light perception could compromise the adaptability of the suprachiasmatic nucleus (SCN) to the diurnal rhythm (27, 28). The SCN regulates the phase shift and secretion levels of melatonin, influencing the sleep-wake cycle. This maladaptation precipitates a desynchronization of sleep-wake cycles, culminating in compromised sleep quality.

Additionally, suboptimal sleep quality in individuals with diminished VA might also stem from dysfunctional photoreceptor cells and associated mechanisms. The rod-cone photoreceptor system, in conjunction with the melanopsin system, plays a pivotal role in mediating the effects of light on sleep (10, 29). This mediation primarily occurs through the intermediation of intrinsically photosensitive retinal ganglion cells (ipRGCs) (10, 29, 30). Ocular pathologies, including DR (31), glaucoma (32), age-related macular degeneration (AMD) (33), and optic disc disorders, can markedly impair sleep quality by detrimentally affecting these cells. Furthermore, the increased likelihood of poor sleep quality continued to be evident in patients with diminished VA, even after adjusting for these ocular conditions. This finding firstly substantiates the robustness of our results. Secondly, it suggests that factors beyond photoreceptor cell damage contribute to the poor sleep quality associated with diminished VA.

VI may affect sleep quality through several pathways. These include reduced outdoor activity (34), increased vigilance due to safety concerns (35, 36), and potentially greater exposure to sleep-disrupting blue light from adaptive devices (37).

In our research, we found a J-shaped relationship between VA and sleep quality. The lowest point of this curve occurred at approximately 0.5 LogMAR. The probability of poor sleep quality increased as VA worsened, up to this point. Beyond 0.5 LogMAR, the probability plateaued. Our study is the first to report this J-shaped association. Currently, no existing studies are available for direct comparison. Even a minor decline in VA can negatively influence sleep quality, even before reaching the threshold of clinical VI.

Specifically, patients with VA ranging from 0.1-0.2 LogMAR demonstrated a 1.7-fold increased likelihood of poor sleep quality compared to those with normal vision. Importantly, this identifies a pre-clinical threshold where mild visual decline compromises sleep quality before meeting formal visual impairment criteria. Our findings have important implications for clinical practice in ophthalmology and optometry. They suggest the need for a more integrated approach to patient care. Eye care professionals should consider screening for sleep issues in high-risk patients with visual impairments. This could facilitate timely referrals when necessary. A proactive strategy would include early interventions such as sleep hygiene education, phototherapy, and referrals to sleep specialists. This approach aligns with the growing focus on preventive care in healthcare systems. Patient education about how visual impairment affects sleep quality offers additional benefits. It may improve adherence to vision care treatments and encourage sleep-enhancing behaviors. Our findings also emphasize the importance of collaboration between eye care professionals and sleep specialists. This is especially valuable during the early stages of vision decline. Previous research supports this integrated approach. A study by Smith et al. (38) showed improved sleep quality in cataract patients after intraocular lens replacement. This highlights the potential benefits of timely intervention for treatable eye conditions.

Our findings also suggest a critical threshold at approximately 0.5 LogMAR, marking the clinical demarcation between non-VI and VI. The 0.5 LogMAR threshold represents a critical functional boundary where individuals typically begin experiencing significant difficulties with daily activities. Beyond this threshold, sleep quality in individuals tends to stabilize across the VA spectrum.

The loss of association beyond the 0.5 LogMAR threshold suggests a fundamental shift in sleep regulation mechanisms. This threshold pattern suggests different underlying mechanisms across the visual acuity spectrum. We propose that individuals without VI may primarily regulate their circadian rhythms through light perception via the eyes. In contrast, those with VI may rely more on non-visual sensory input.

Specifically, for individuals with better visual acuity, circadian rhythm regulation appears to depend primarily on the traditional rod-cone photoreceptor system, where progressive visual decline leads to proportional sleep quality deterioration. However, beyond the 0.5 LogMAR threshold, a fundamental shift occurs toward alternative regulatory pathways. Patients with VI may transition to greater reliance on the intrinsically photosensitive retinal ganglion cell-melanopsin pathway for circadian photoentrainment, which maintains basic rhythm regulation even in profound visual loss (39, 40). Additionally, non-visual sensory compensation through auditory (41) and thermal (42) environmental cues may partially replace diminished visual input. However, we don’t fully understand how the rod-cone and melanopsin systems work together at different levels of visual acuity. This gap in knowledge highlights the necessity for more comprehensive experimental research to corroborate these hypotheses. Given the dramatically varying effects of light therapy on sleep across individuals (43), gaining a deeper insight into this interplay is also crucial for refining the application of phototherapeutic interventions. Sleep interventions for people with VI might therefore need to differ from those with mild VI.

While our findings suggest that visual acuity may influence sleep quality, we must also consider the possibility of reverse or bidirectional causality. Poor sleep quality could potentially affect visual function through several biological mechanisms. Sleep deprivation and poor sleep quality increase oxidative stress and inflammatory markers such as C-reactive protein and interleukins, which may contribute to retinal damage over time (44). Chronic sleep disturbances have been associated with impaired vascular regulation and increased risk of microvascular complications (8), which could adversely affect the retinal microvasculature. The cross-sectional nature of our study prevents definitive causal inference, and longitudinal studies are necessary to establish the temporal relationship between visual decline and sleep disturbances.

Findings regarding the correlation between VA and sleep duration present a contentious landscape. The Korea National Health and Nutrition Examination Survey (KNHNES) (45) posited that VI correlates with both short and long sleep durations. However, Peltzer et al. (14) reported no significant association between these variables. Our findings support the latter conclusion. We found no relationship between VA and sleep duration when analyzing VA as a segmented variable. Such findings imply that deep sleep duration, rather than total sleep duration, may more significantly influence sleep quality. Relying solely on sleep duration as a metric is insufficient for assessing overall sleep health.

Several methodological strengths enhance the reliability of our findings. Our large sample size enabled comprehensive analysis across the visual acuity spectrum in real-world settings, supporting sophisticated statistical approaches including restricted cubic spline modeling and multiple sensitivity analyses. Importantly, we incorporated depression adjustment based on emerging evidence of its mediating role in sleep-health relationships, ensuring more accurate estimation of the direct association between visual acuity and sleep quality (46).

Our study has several limitations. First, its observational nature prevents establishing causal relationships. Second, we did not screen for conditions such as obstructive sleep apnea, REM sleep disorders, or measure melatonin levels. This limited our comprehensive assessment of sleep health. Moreover, sleep duration was assessed using PSQI Component 3, which relies on a single-item self-report measure susceptible to recall bias and may not capture night-to-night variability in sleep patterns. Third, we excluded individuals without VA records, which could introduce selection bias. We chose not to use multiple imputation for these missing data because individuals without VA tests often have poorer vision. This violates the “missing at random” assumption required for imputation. We cannot rule out unmeasured confounding factors such as socioeconomic conditions and genetic variation. However, our homogeneous study population and extensive data on known risk factors likely reduced potential confounding. Finally, our focus on patients with diabetes mellitus limits the generalizability of our results to broader populations.

In conclusion, our study elucidates a J-shaped association between VA and sleep quality within a substantial community-based population. We observed that the risk of poor sleep quality commences even with a slight decline in VA. Notably, the positive relationship between VA and sleep quality diminished in individuals with VI. This insight into the independent role of VA on sleep is pivotal, offering significant implications for demystifying the intricate interplay among ambient light exposure, photoreceptor cell functioning, and the regulation of sleep.

## Data Availability

The raw data supporting the conclusions of this article will be made available by the authors, without undue reservation.

## Supplementary material

Supplementary Table 1 Sensitivity analysis for the association of visual acuity with sleep quality and duration in the FS-DIRECT study. OR, odds ratio; BMI, body mass index; CKD, chronic kidney disease; DR, diabetic retinopathy; AMD, age-related macular degeneration. ^a^ The odds ratios for poor sleep quality vs. good sleep quality, short sleep duration vs. normal sleep duration, and long sleep duration vs. normal sleep duration in logistic regression. ^b^Model 2: the same as model 2 in the main analysis, with adjustment for age, sex, marital status, educational level, duration of diabetes, and coronary heart disease.

Supplementary Figure 1 Visual Acuity and Its Association with Sleep Outcomes Across Selected Subgroups Odds ratios (ORs) with 95% confidence intervals (CIs) are displayed per 0.2 LogMAR unit increase in visual acuity. The statistical model in the stratified analysis was the same as model 2, adjusted for age, sex, marital status, educational level, duration of diabetes, hemoglobin A1c, body Mass Index, and the prevalence of medical conditions like stroke, chronic kidney disease, hyperlipidemia and coronary heart disease.
