# Supplementary material for: Association of visual acuity with sleep quality and sleep duration in patients with type 2 diabetes: evidence from a cross-sectional analysis of the Fushun Diabetic Retinopathy Study
Source: Front Psychiatry. 2025 Jul 17;16:1521347. doi: 10.3389/fpsyt.2025.1521347 (PMC12310673; doi:10.3389/fpsyt.2025.1521347)
Supplement: Supplementary Table 1 — Sensitivity analysis for the association of visual acuity with sleep quality and duration in the FS-DIRECT study. OR, odds ratio; BMI, body mass index; CKD, chronic kidney disease; DR, diabetic retinopathy; AMD, age-related macular degeneration. a The odds ratios for poor sleep quality vs. good sleep quality, short sleep duration vs. normal sleep duration, and long sleep duration vs. normal sleep duration in logistic regression. bModel 2: the same as model 2 in the main analysis, with adjustment for age, sex, marital status, educational level, duration of diabetes, and coronary heart disease. [file Table1.docx]

**Supplementary Table1.** Sensitivity Analysis for the Association of Visual Acuity with Sleep Quality and Duration in the FS-DIRECT Study

|  |  | **OR (95% CI)** ^a^ | | | | | | |
| --- | --- | --- | --- | --- | --- | --- | --- | --- |
| **Models** | **No.** | **Group 1: (<0 LogMAR)** | **Group 2: (0-0.1 LogMAR)** | **Group 3: (0.1-0.2 LogMAR)** | **Group 4: (0.2-0.3 LogMAR)** | **Group 5: (0.3-0.5 LogMAR)** | **Group 6: (≥0.5 LogMAR)** | **Per 0.2 LogMAR increase** |
| **Poor sleep quality** |  |  |  |  |  |  |  |  |
| Model 2 ^b^ | 1263 | 1 [Reference] | 1.167 (0.864, 1.581) | 1.752 (1.072, 2.861) | 2.006 (1.130, 3.574) | 1.475 (0.868, 2.497) | 1.698 (1.111, 2.598) | 1.089 (1.017, 1.167) |
| Model 2, with multiple imputation | 1284 | 1 [Reference] | 1.168 (0.865, 1.577) | 1.695 (1.043, 2.756) | 2.029 (1.151, 3.575) | 1.513 (0.900, 2.543) | 1.702 (1.117, 2.594) | 1.090 (1.019, 1.166) |
| Model 2, + employment, income | 1263 | 1 [Reference] | 1.177 (0.871, 1.595) | 1.732 (1.059, 2.832) | 2.022 (1.138, 3.604) | 1.491 (0.877, 2.525) | 1.709 (1.117, 2.616) | 1.090 (1.018, 1.168) |
| Model 2, + smoking status, and alcohol intake | 1263 | 1 [Reference] | 1.177 (0.870, 1.596) | 1.811 (1.105, 2.967) | 2.013 (1.133, 3.593) | 1.506 (0.884, 2.554) | 1.743 (1.137, 2.675) | 1.093 (1.020, 1.171) |
| Model 2, + treatment of diabetes | 1201 | 1 [Reference] | 1.168 (0.857, 1.596) | 1.731 (1.037, 2.885) | 1.986 (1.111, 3.562) | 1.422 (0.825, 2.437) | 1.727 (1.119, 2.670) | 1.095 (1.021, 1.176) |
| Model 2, + anxiety | 1263 | 1 [Reference] | 1.153 (0.852, 1.565) | 1.642 (0.999, 2.698) | 2.004 (1.124, 3.587) | 1.450 (0.849, 2.466) | 1.654 (1.077, 2.543) | 1.085 (1.013, 1.164) |
| Model 2, + DR, AMD, glaucoma, and optic nerve disorders | 1263 | 1 [Reference] | 1.165 (0.862, 1.578) | 1.757 (1.074, 2.871) | 1.972 (1.111, 3.515) | 1.488 (0.875, 2.520) | 1.713 (1.120, 2.621) | 1.090 (1.018, 1.168) |
| **Short sleep duration** |  |  |  |  |  |  |  |  |
| Model 2 ^b^ | 1149 | 1 [Reference] | 1.181 (0.894, 1.562) | 1.371 (0.881, 2.135) | 1.541 (0.895, 2.661) | 1.465 (0.875, 2.459) | 1.177 (0.783, 1.770) | 1.003 (0.940, 1.069) |
| Model 2, with multiple imputation | 1168 | 1 [Reference] | 1.170 (0.888, 1.543) | 1.351 (0.872, 2.094) | 1.561 (0.915, 2.663) | 1.454 (0.875, 2.417) | 1.137 (0.758, 1.704) | 0.997 (0.935, 1.062) |
| Model 2, + employment, income | 1149 | 1 [Reference] | 1.181 (0.894, 1.562) | 1.372 (0.881, 2.137) | 1.541 (0.896, 2.661) | 1.465 (0.874, 2.459) | 1.177 (0.782, 1.769) | 1.002 (0.940, 1.069) |
| Model 2, + smoking status, and alcohol intake | 1149 | 1 [Reference] | 1.176 (0.890, 1.556) | 1.378 (0.884, 2.151) | 1.531 (0.888, 2.648) | 1.459 (0.870, 2.452) | 1.181 (0.784, 1.778) | 1.003 (0.941, 1.070) |
| Model 2, + treatment of diabetes | 1090 | 1 [Reference] | 1.254 (0.939, 1.676) | 1.487 (0.937, 2.364) | 1.653 (0.953, 2.875) | 1.581 (0.929, 2.697) | 1.318 (0.866, 2.005) | 1.014 (0.949, 1.084) |
| Model 2, + anxiety | 1149 | 1 [Reference] | 1.177 (0.890, 1.557) | 1.392 (0.890, 2.178) | 1.553 (0.899, 2.693) | 1.400 (0.831, 2.362) | 1.201 (0.796, 1.810) | 1.004 (0.941, 1.071) |
| Model 2, + DR, AMD, glaucoma, and optic nerve disorders | 1149 | 1 [Reference] | 1.182 (0.895, 1.564) | 1.369 (0.880, 2.133) | 1.547 (0.899, 2.673) | 1.465 (0.874, 2.459) | 1.174 (0.780, 1.765) | 1.002 (0.940, 1.069) |
| **Long sleep duration** |  |  |  |  |  |  |  |  |
| Model 2 ^b^ | 748 | 1 [Reference] | 1.259 (0.755, 2.142) | 1.180 (0.483, 2.675) | 0.845 (0.229, 2.478) | 2.265 (0.971, 5.093) | 1.482 (0.727, 2.971) | 1.019 (0.912, 1.126) |
| Model 2, with multiple imputation | 761 | 1 [Reference] | 1.210 (0.725, 2.021) | 1.122 (0.483, 2.606) | 0.794 (0.249, 2.534) | 2.127 (0.940, 4.814) | 1.487 (0.748, 2.957) | 1.029 (0.931, 1.139) |
| Model 2, + employment, income | 748 | 1 [Reference] | 1.257 (0.754, 2.140) | 1.187 (0.486, 2.692) | 0.840 (0.227, 2.464) | 2.256 (0.967, 5.072) | 1.476 (0.724, 2.958) | 1.018 (0.912, 1.125) |
| Model 2, + smoking status, and alcohol intake | 748 | 1 [Reference] | 1.238 (0.742, 2.108) | 1.156 (0.471, 2.630) | 0.854 (0.231, 2.503) | 2.104 (0.895, 4.764) | 1.445 (0.709, 2.896) | 1.015 (0.909, 1.122) |
| Model 2, + treatment of diabetes | 717 | 1 [Reference] | 1.283 (0.762, 2.210) | 1.171 (0.457, 2.748) | 0.848 (0.228, 2.508) | 2.475 (1.051, 5.636) | 1.728 (0.839, 3.509) | 1.034 (0.926, 1.144) |
| Model 2, + anxiety | 748 | 1 [Reference] | 1.249 (0.748, 2.130) | 1.152 (0.468, 2.631) | 0.774 (0.208, 2.289) | 2.137 (0.907, 4.839) | 1.365 (0.663, 2.761) | 1.009 (0.902, 1.116) |
| Model 2, + DR, AMD, glaucoma, and optic nerve disorders | 748 | 1 [Reference] | 1.259 (0.754, 2.146) | 1.115 (0.455, 2.539) | 0.840 (0.226, 2.487) | 2.267 (0.966, 5.131) | 1.460 (0.717, 2.925) | 1.018 (0.911, 1.125) |

Abbreviations: OR, odds ratio; BMI, body mass index; CKD, chronic kidney disease; DR, diabetic retinopathy; AMD, age-related macular degeneration.

^a^ The odds ratios for poor sleep quality vs. good sleep quality, short sleep duration vs. normal sleep duration, and long sleep duration vs. normal sleep duration in logistic regression.

^b^ Model 2: the same as model 2 in the main analysis, with adjustment for age, sex, marital status, educational level, duration of diabetes, and coronary heart disease.
