# Supplementary figures and images for: Association of visual acuity with sleep quality and sleep duration in patients with type 2 diabetes: evidence from a cross-sectional analysis of the Fushun Diabetic Retinopathy Study
Source: Front Psychiatry. 2025 Jul 17;16:1521347. doi: 10.3389/fpsyt.2025.1521347 (PMC12310673; doi:10.3389/fpsyt.2025.1521347)

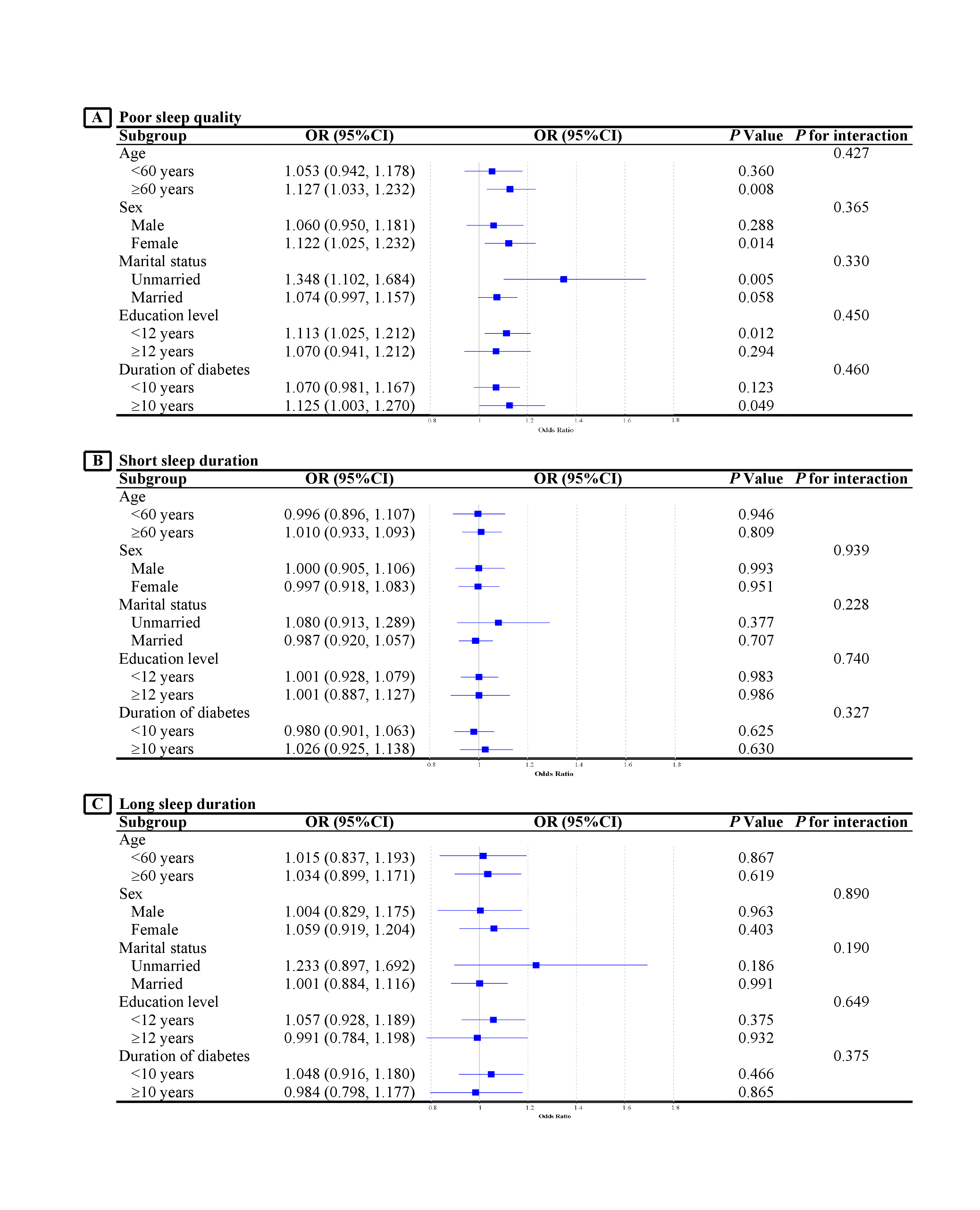

Supplement: Supplementary Figure 1 — Visual Acuity and Its Association with Sleep Outcomes Across Selected Subgroups Odds ratios (ORs) with 95% confidence intervals (CIs) are displayed per 0.2 LogMAR unit increase in visual acuity. The statistical model in the stratified analysis was the same as model 2, adjusted for age, sex, marital status, educational level, duration of diabetes, hemoglobin A1c, body Mass Index, and the prevalence of medical conditions like stroke, chronic kidney disease, hyperlipidemia and coronary heart disease. [file Image1.jpg]
